# Vibration Sensor-Based Bearing Fault Diagnosis Using Ellipsoid-ARTMAP and Differential Evolution Algorithms

**DOI:** 10.3390/s140610598

**Published:** 2014-06-16

**Authors:** Chang Liu, Guofeng Wang, Qinglu Xie, Yanchao Zhang

**Affiliations:** Key Laboratory of Mechanism Theory and Equipment Design of Ministry of Education, Tianjin University, Tianjin 300072, China; E-Mails: lc8542969@163.com (C.L.); xql246888@126.com (Q.X.); zhangyanchao1991@126.com (Y.Z.)

**Keywords:** fault classification, rolling element bearing, Ellipsoid-ARTMAP network, differential evolution

## Abstract

Effective fault classification of rolling element bearings provides an important basis for ensuring safe operation of rotating machinery. In this paper, a novel vibration sensor-based fault diagnosis method using an Ellipsoid-ARTMAP network (EAM) and a differential evolution (DE) algorithm is proposed. The original features are firstly extracted from vibration signals based on wavelet packet decomposition. Then, a minimum-redundancy maximum-relevancy algorithm is introduced to select the most prominent features so as to decrease feature dimensions. Finally, a DE-based EAM (DE-EAM) classifier is constructed to realize the fault diagnosis. The major characteristic of EAM is that the sample distribution of each category is realized by using a hyper-ellipsoid node and smoothing operation algorithm. Therefore, it can depict the decision boundary of disperse samples accurately and effectively avoid over-fitting phenomena. To optimize EAM network parameters, the DE algorithm is presented and two objectives, including both classification accuracy and nodes number, are simultaneously introduced as the fitness functions. Meanwhile, an exponential criterion is proposed to realize final selection of the optimal parameters. To prove the effectiveness of the proposed method, the vibration signals of four types of rolling element bearings under different loads were collected. Moreover, to improve the robustness of the classifier evaluation, a two-fold cross validation scheme is adopted and the order of feature samples is randomly arranged ten times within each fold. The results show that DE-EAM classifier can recognize the fault categories of the rolling element bearings reliably and accurately.

## 1. Introduction

Fault diagnosis, which includes fault detection and isolation (FDI) [1,2], fault tolerant control (FTC) [3,4] and fault classification [5,6], plays an important role in automation systems, process engineering and mechanical equipment. In these research fields, fault recognition of rolling element bearings has attracted more and more attention of many researchers. As one of the most critical mechanical components, it has found wide applications in rotating machinery such as motors, pumps, compressors and wind power generators, *etc.* However, its defects may cause malfunctions or even lead to catastrophic failure of the rotating machinery if not diagnosed in time. The available studies have shown that bearing faults account for 40% of all machinery failures [7,8]. Therefore, effective fault monitoring is necessary for reducing maintenance costs and avoiding unscheduled downtime. Different from some diagnosing methods used in control systems and industrial processes, data driven models are commonly adopted to realize condition monitoring and fault diagnosis of rolling element bearings [5,6,9]. In comparison with the model driven method used in [1–4], the advantage of data-based modeling lies that the model can be built without understanding the complex internal mechanics of the mechanical system. For rolling element bearings, an accurate analytic dynamic model to depict the characteristic of rolling element bearings is hard to construct because there are so many sub-parts which include inner race, outer race, elements, *etc.* Therefore, data driven models based on historic data is preferred in real industrial applications and its robustness mainly depends on effective signal preprocessing, feature extraction and statistical modeling strategies.

In the past two decades, various kinds of pattern recognition techniques such as artificial neural networks (ANNs) [10–12] and support vector machines (SVMs) [13–15] have been successfully applied to classifying bearing faults. These methods have been proven to be capable of obtaining favorable classification accuracy in some research areas [5]. However, in real applications, the useful information is usually contaminated by external noise or internal load variation. In such cases, the classifiers based on the above methods are liable to cause over-fitting phenomena and deteriorate the classification performance [6]. At the same time, these methods seek to depict the boundary of the classifier as the function of several support vectors or network weight values, which makes it hard to characterize the complex decision boundary if the spatial distributions of the training samples is disperse and irregular.

In this paper, Ellipsoid-ARTMAP (EAM), which is based on adaptive resonance theory (ART) [16], is presented to realize the task of classifying complex samples due to its strong local and distributed representation ability [17–19]. Unlike Radius Basis Function (RBF) neural network, which is utilized to estimate the probability density of the data streams and judge the occurrence of the fault by comparing the probability density of the current data with the previous one [20], EAM is mainly proposed to depict the spatial distribution of the sample data for each category. One major characteristic of EAM is that distributed hyper-ellipsoid clusters are utilized to realize the geometric representation of each category. Therefore, the decision boundary of the dispersive samples can be depicted accurately and flexibly. Another is that smoothing operation is performed during the updating process of these ellipsoid nodes, which makes it insensitive to noise and can avoid over-fitting. Xu *et al.* [21] applied the EAM network to classify the gene expression data generated by DNA microarray experiments. They claimed that a good performance can be achieved. Anagnostopoulos *et al.* [22,23] utilized EAM for the Circle-in-a-Square classification problem. The authors proved that EAM demonstrated better ability in clustering and classification tasks than other ARTMAP networks. However, it can be also demonstrated that the performance of EAM network is greatly affected by its parameter selection strategy. In fact, there are four important parameters that need to be determined a *priori*: vigilance parameter, learning rate, effective diameter and ratio of minor-to-major axes lengths. Each parameter exerts a significant impact on the performance of the EAM classifier. Currently, the selection of these parameters mainly depends on empirical selection or trial and error methods, which inevitably bring inaccuracy and uncertainty to the classification performance. To solve this problem, a differential evolution (DE) algorithm is adopted in this paper to optimize the network parameters of EAM. One reason for selecting DE is that it has strong global optimization ability and imposes no demands for the analytical mathematical expression of the fitness function, which is paramount for EAM parameter optimization because such an equation does not exist during the classification process. In addition, the DE has several advantages: compared with traditional evolutionary optimization algorithms, its fewer control parameters make DE simpler and more straightforward to implement. Meanwhile, the differential operation of the candidate parameter vectors optimization makes it more effective to find a global optimum when handling large scale and multi-objective optimization problems [24]. Up to now, DE algorithm has been used in many applications such as system design [25], partitioned clustering [26] and classification tasks [27].

Based on the DE optimization algorithm and EAM classifier, a vibration sensor-based fault diagnosis system is constructed. In this framework, vibration signals were collected and wavelet packet decomposition (WPD) is introduced to extract features to characterize the fault related information. Meanwhile, the minimum-redundancy maximum-relevancy (mRMR) feature selection method is employed to decrease the redundancy and irrelevancy of these features. Finally, the EAM classifier is constructed based on the selected salient features to realize fault classification of rolling element bearings and DE is integrated with EAM to optimize the classifier parameters during the training process. In order to get satisfactory accuracy without lowering the recognition speed, both the classification accuracy and number of nodes are adopted as the fitness functions of the DE algorithm. Meanwhile, a new exponential selection criterion is presented to balance the influence of both functions and choose the final parameters. To verify the effectiveness of the DE-EAM classifier, four kinds of faulty vibration signals (normal, inner race fault, outer race fault and ball fault) were collected from a test rig. Meanwhile, two-fold cross validation and random order strategies are adopted to evaluate the performance of classifier robustly and accurately. The final results of the bearing faults diagnosis show that DE-EAM method can accurately recognize the fault categories.

The rest of the paper is organized as follows: in Section 2, the principles of the EAM classifier and DE optimization algorithm are explained, respectively. In Section 3, the framework of the DE-EAM- based monitoring system is presented. Moreover, the principle of WPD feature extraction and mRMR feature selection are discussed in detail. In Section 4, the effectiveness of the proposed method is verified by classification of four kinds of bearing faults under a cross validation and random order strategy. Some useful conclusions are drawn in Section 5.

## 2. Principle of the DE-EAM Algorithm

### 2.1. Ellipsoid-ARTMAP (EAM)

#### 2.1.1. Network Structure

**Figure 1.**
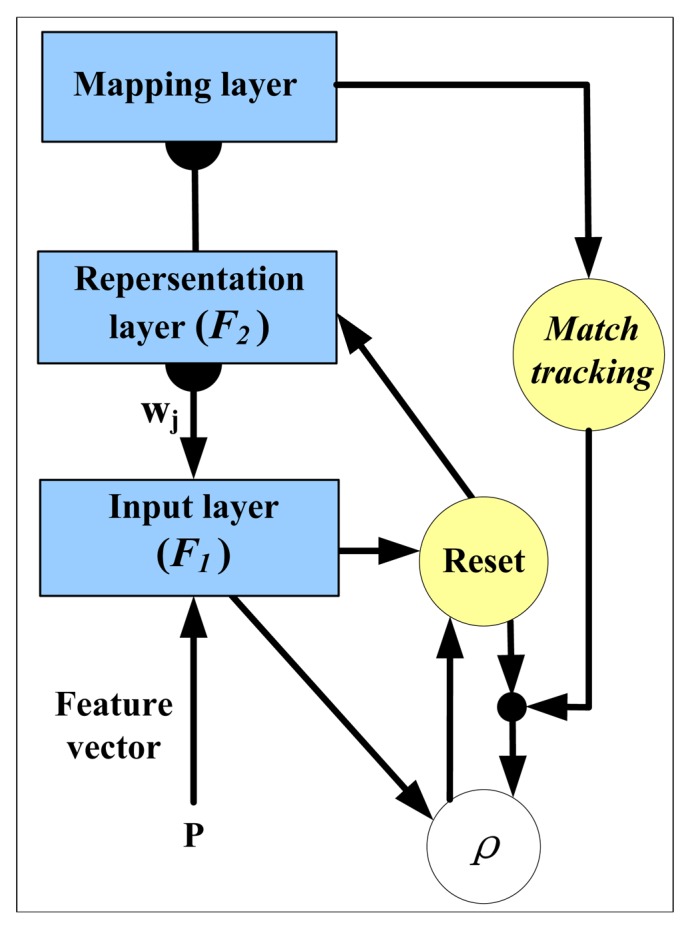
The framework of the Ellipsoid-ARTMAP network.

Figure 1 shows the framework of EAM network. It can be seen that a typical EAM structure consists of three layers: input layer F_1_, representation layer F_2_ and mapping layer F_3_. F_1_ and F_2_ layer are connected by the template vector **w**_j_, which is utilized to encode the input sample into the *j*th node in F_2_ layer. If the node passed the vigilance test, the commitment test is then applied on these nodes and the winner nodes are mapped into the F_3_ layer so as to get the classification results. During the training process, a match tracking (MT) process [16] will be invoked when the output of F_3_ layer is incorrect.

#### 2.1.2. Node Representation

**Figure 2.**
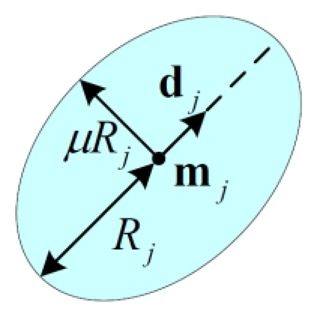
Two-dimensional representation of an EAM node.

Figure 2 shows a two-dimensional representation of an EAM node. Each node *j* is described by a template vector **w**_j_=[**m**_j_,**d**_j_,*R*_j_] where **m**_j_ is the center of hyper-ellipsoid, **d**_j_ is the direction vector of node, which coincides with the direction of the hyper-ellipsoid's major axis, and *R*_j_ is called the radius of the node, which equals half the length of the major axis.

#### 2.1.3. Distance Calculation

The distance between an input sample **x** and the node *j* is given by [16]:

(1) $$\text{dis}(\text{x}|{\text{w}}_{\text{j}})=\max\left\{{\left\|\text{x}-{\text{m}}_{\text{j}}\right\|}_{C_{j}},R_{\text{j}}\right\}-R_{\text{j}}$$

where ‖**x**–**m**_j_‖*_C_*_j_ is the distance between a sample **x** and the center **m**_j_ of the node *j*, which can be expressed as [22]:

(2) $${\left\|\text{x}-{\text{m}}_{j}\right\|}_{c_{j}}=\{\begin{array}{cc} \frac{1}{\mu }\sqrt{{\left\|\text{x}-{\text{m}}_{j}\right\|}_{2}^{2}-(1-\mu^{2}){;d_{j}^{T}(\text{x}-{\text{m}}_{j})=}^{2}} & \text{if}\;{\text{d}}_{j}\neq 0\\ \left\|\text{x}-{\text{m}}_{j}\right\| & \text{if}\;{\text{d}}_{j}\neq 0 \end{array}$$

where ‖·‖_2_ means the usual Euclidian (L2) norm, *μ* is the ratio of minor-to-major axes lengths and *C*_j_ is node shape matrix. As shown in Figure 2, the shaded areas denote the representation regions of the node *j*, which are the set of points that satisfy the following condition:

(3) $$\text{dis}(\text{x}|{\text{w}}_{j})=0\;\Rightarrow \;{\left\|\text{x}-{\text{m}}_{j}\right\|}_{c_{j}}\leq R_{j}$$

#### 2.1.4. Vigilance Test (VT) and Commitment Test (CT)

For an input sample **x**, the node *j* features two important values which are called the category match function (CMF) value ρ*(**w**_j_|**x**) and the category choice function (CCF) value T*(**w**_j_|**x**) [16]:

(4) $$\rho^{*}({\text{w}}_{\text{j}}|\text{x})=1-\frac{R_{\text{j}}+\max\left\{{\left\|\text{x}-{\text{m}}_{\text{j}}\right\|}_{C_{j}},R_{\text{j}}\right\}}{D_{m}}$$

(5) $$T^{*}({\text{w}}_{\text{j}}|\text{x})=\frac{D-R_{\text{j}}-\max\left\{{\left\|\text{x}-{\text{m}}_{\text{j}}\right\|}_{C_{j}},R_{\text{j}}\right\}}{D_{m}-2*R_{\text{j}}+a}$$

where *D_m_* is called effective diameter, *a* is the choice parameter. The choice parameter *a* is a really small positive value which has no obvious influence on EAM performance. In this paper, *a* is selected as 0.001. Usually, uncommitted nodes have a constant CCF value *T_u_* = *D_m_*/(2*D_m_w+a*). The parameter *ω* is usually chosen as *ω*≥0.5 to ensure the stability of EAM. If the CMF value and CCF value of the node *j* for the given input sample **x** are larger than initial vigilance parameter *ρ̄* and *T_u_*, this node is called a committed node. These two comparison processes are called the vigilance test (VT) and commitment test (CT), respectively.

#### 2.1.5. Node Updating

During the training process, the committed node is updated according to the input sample. That is, its template vector **w**_j_ is recalculated according to the formula below [23]:

(6) $$R_{\text{j}}^{\text{new}}=R_{\text{j}}^{\text{old}}+\frac{\gamma }{2}(\max\left\{R_{\text{j}}^{\text{old}},{\left\|\text{x}-{\text{m}}_{\text{j}}^{\text{old}}\right\|}_{C_{j}^{\text{old}}}\right\}-R_{\text{j}}^{\text{old}})$$

(7) $${\text{m}}_{\text{j}}^{\text{new}}={\text{m}}_{\text{j}}^{\text{old}}+\frac{\gamma }{2}\left(1-\frac{\max\{R_{\text{j}}^{\text{old}},{\left\|\text{x}-{\text{m}}_{\text{j}}^{\text{old}}\right\|}_{C_{j}^{\text{old}}}\}}{{\left\|\text{x}-{\text{m}}_{\text{j}}^{\text{old}}\right\|}_{C_{j}^{\text{old}}}}\right)(\text{x}-{\text{m}}_{\text{j}}^{\text{old}})$$

(8) $${\text{d}}_{\text{j}}=\frac{{\text{x}}_{\left(2\right)}-{\text{m}}_{\text{j}}}{{\left\|{\text{x}}_{\left(2\right)}-{\text{m}}_{\text{j}}\right\|}_{2}}$$

where *γ*∈(0,1] denotes the learning rate. If *γ* is selected as 1, it means that the EAM has the ability of fast learning. **x**_(2)_ represents the second sample encoded by the node *j*.

**Figure 3.**
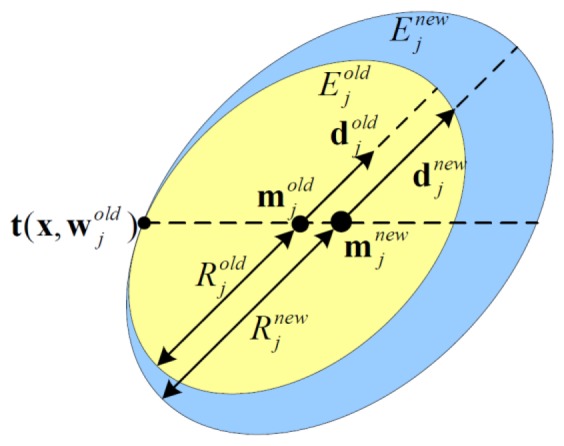
The updating process of a two-dimensional EAM node.

A two-dimensional description of node updating based on Equations (6)–(8) is shown in Figure 3. It can be shown that the EAM node can only grow in size and never be destroyed during the training process. The node's new representation region is a minimum hyper-ellipsoid which contains both the old region and the new sample to be encoded. In addition, when the EAM node's direction vector is set, it will not change during the node updating process. Besides that, due to the minimum hyper-ellipsoid is used to characteristic the old region and the new pattern, the *E^new^* and *E^old^* can only touch at one point.

#### 2.1.6. Training and Classification

**Figure 4.**
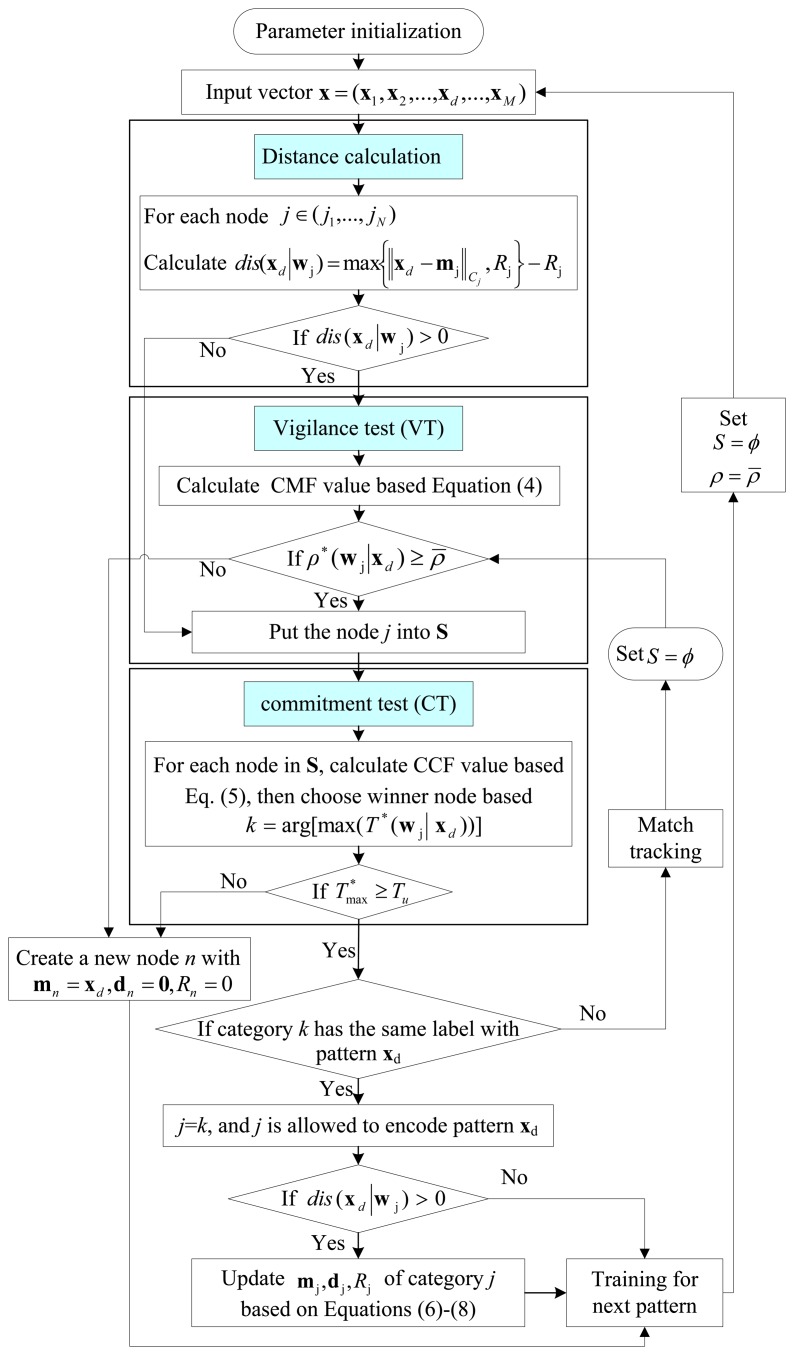
Flowchart of EAM training.

The complete training process of the EAM classifier is given in the following steps:

Step 1 The network parameters are initialized.
Step 2 The distance calculation is started based on Equation (3) to judge whether the training sample belongs to the representation region of the node *j* or not. If not, the input sample will undergo the VT. That is to say, the value of ρ*(**w**_j_|**x**) larger than $\bar{\rho }$ will be selected into a candidate set **S**, otherwise, it will be removed.
Step 3 CCF value of all member nodes in **S** is calculated and compared with *T_u_*. The nodes that cannot pass the CT will be removed from **S**. If no nodes pass the VT and CT (here, **S** is empty), new node will be created and initiated.
Step 4 If the set **S** is non-empty after the VT and CT, the labels of the training sample and the chosen node *j* will be used for comparison. If the selected node *j* shares the same label with the input sample, the template vector **w**_j_ is updated. If the label is not correct, the match tracking (MT) process will be activated. The flowchart of the EAM training is given in Figure 4.

The EAM classification is similar to the training process. The only difference is that the MT process is neglected if the EAM is used for classification. If all nodes are not committed, the output of the EAM classifier is −1, which denotes that an abnormal result appears.

As mentioned above, the following four parameters have an important influence on the performance of the EAM classifier: the effective diameter *D_m_*, the ratio of minor-to-major axes lengths *μ*∈(0,1], the vigilance parameter *ρ̄* ∈ [0,1] and the learning rate γ∈(0,1]. Vigilance parameter *ρ̄* is a threshold value which is a necessary condition for judging whether the input belongs to the corresponding node or not. Learning rate γ assure that EAM has a flexible learning ability range from slow learning to fast learning. Effective diameter *D_m_* is always chosen to ensure the stability of EAM. Ratio of minor-to-major axes lengths *μ* determines the shape of the hyper-ellipsoid node. To improve the accuracy of the classifier, differential evolution (DE) algorithm is introduced to obtain the optimal combination of these parameters. The principle of DE is depicted in the following section.

### 2.2. Differential Evolution (DE)

As a member of the evolutionary optimization algorithm class, the DE algorithm uses three classical operators: mutation, crossover and selection, to generate trial parameter vectors and select the final solution with best fitness [28]. The main advantage of evolutionary algorithms is their global search abilities with no risk of falling into the local minimum region. In contrast, the gradient-based local research algorithm is not suitable for optimizing the EAM network structure [29,30] because the analytical mathematical expression of the objective function is not available. Moreover, although there are many variants, the traditional DE/rand/1/bin algorithm is still adopted in this paper because of its maturity, robustness and fast convergence features [31–33].

**Figure 5.**
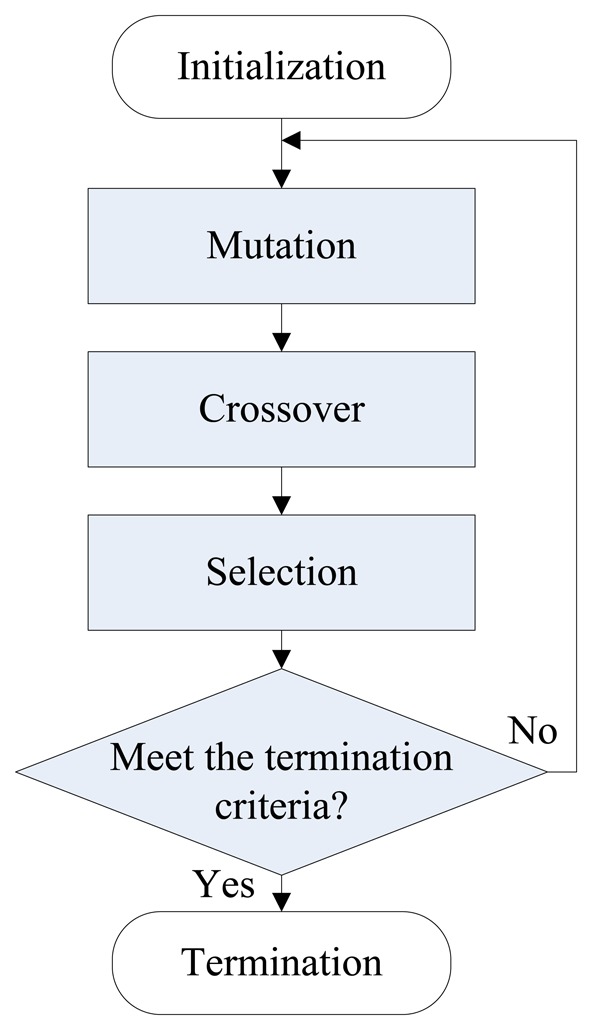
Flowchart of DE iterative optimization.

Each generation in DE comprises *N_p_* populations. The population is expressed as **Y***_i,g_*. i∈(1, …*N_p_*) is the index of population and *g* represents the number of generations. Each population contains parameters that need to be optimized. Here in this paper, four parameters of the EAM network need to be optimized, that is to say, **Y**_*i,g*_ = { *D_m_*,*μ*, *ρ̄*,*γ*}. The main steps of DE algorithm are given as follows:

Step 1 Initialization
  Set the boundaries of the optimized parameters and initialize their value in the first generation according to a uniform probability distribution.
Step 2 Mutation
  Mutation operation is to add a vector differential to a population vector of individuals. For each vector **Y***_i,g_*, the mutant vector is formed based on the following rule:
  (9) $${\text{V}}_{i,g+1}={\text{Y}}_{r1,g}+F\left({\text{Y}}_{r2,g}-{\text{Y}}_{r3,g}\right)$$
  where, **Y***_r_*_1_*_,g_*, **Y***_r_*_2_*_,g_* and **Y***_r_*_3_*_,g_* are selected from the populations randomly. The indices *r*1, *r*2 and *r*3 are different from each other and also differ with the current individual *i* (*i.e., r*1≠*r*2≠*r*3≠*i*). *F*∈[0,1] is a scaling factor which controls the amplitude of the difference vector (**Y***_r2,g_*–**Y***_r3,g_*).
Step 3 Crossover
  After the mutation operation, DE utilizes crossover operation to build the trial vectors. Specially, the elements from the parent vector **Y***_j,i,g_* will be combined with **V***_j,i,g_* so as to produce the new element **U***_j,i,g_*_+1_, which is defined as:
  (10) $${\text{U}}_{j,i,g+1}=\{\begin{array}{ll} {\text{V}}_{j,i,g+1} & \text{if}({\text{rand}}_{j}<C)\;\text{or}\;(\tau =j)\\ {\text{Y}}_{j,i,g} & \text{otherwise} \end{array}$$
  where, *j* = 1,2,…*V*, *rand_j_*∈[0,1] is a random number, *C*∈[0,1] is a predefined crossover parameter, *τ*∈(1,2,…,*V*) is a randomly selected index. *τ* = *j* enables at least one of the parameters in the offspring to be different from their parent.
Step 4 Selection
  Selection operation is utilized to choose the better offspring based on the follow equation:
  (11) $${\text{Y}}_{i,g+1}=\{\begin{array}{ll} {\text{U}}_{i,g+1} & \text{if}\;f({\text{U}}_{i,g+1})\leq \;f({\text{Y}}_{i,g})\\ {\text{Y}}_{i,g} & \text{otherwise} \end{array}$$
  where, *f*(*) denotes the fitness function. It can be seen that the solution vector with better fitness value is chosen as the new generation. Obviously, the selection process guarantees that the population fitness is either improving or at least maintaining the best values so far.
Step 5 Termination
  The process of the mutation, crossover and selection is repeated until the maximum generation number *g*_max_ is satisfied. The selection of *g*_max_ depends on the trend of the fitness value fluctuation. Generally, the fitness function tends to be stable with the proceeding of the iteration process. The flowchart of DE is given in Figure 5.

## 3. Diagnosis System Based on DE-EAM

### 3.1. Framework

**Figure 6.**
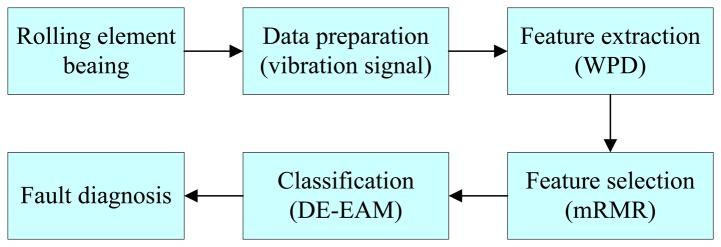
Architecture of bearing fault diagnosis system.

The framework of the DE-EAM based fault diagnosis system is shown in Figure 6. It can be seen that the whole system is composed of four parts: (1) data collection; (2) feature extraction; (3) feature selection; (4) fault classification. Vibration signals from different bearing fault types are collected and Wavelet Packet Decomposition (WPD) is adopted to extract the characteristic features. Then, mRMR method is used to decrease the redundancy and irrelevancy of the original features. Finally, the EAM classifier is constructed to perform bearing fault classification and DE algorithm is used to optimize the network parameters of the EAM classification. The detailed description of each part is given in the sections that follow.

### 3.2. Data Collection

Vibration analysis, due to its simplicity and effectiveness, has been wildly used in bearing fault diagnosis [34–36]. Konar and Chattopadhyay [14] have claimed that the vibration response is the most reliable technique to detect and diagnose the localized defects. In this paper, vibration signals were collected and used to depict the dynamic characteristic of rolling element bearings. As well known, the vibration signal contains both the low-frequency impact and high-frequency structural information. Therefore, the WPD algorithm is adopted in the following section to extract the complete characteristics of the rolling element bearings.

### 3.3. Feature Extraction

**Figure 7.**
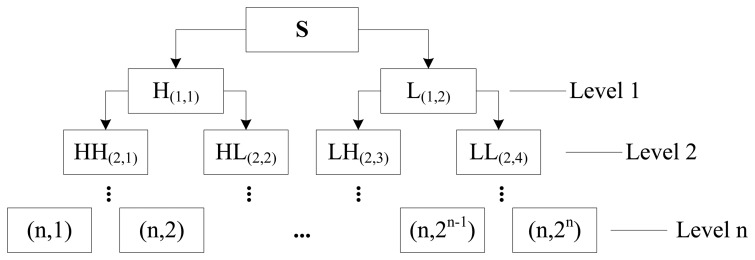
High and low frequency decomposition of vibration signals based on WPD.

WPD is a kind of multi-resolution signal processing algorithm. The main characteristic of WPD is that both the low-frequency and high-frequency information are decomposed simultaneously. The decomposition structure of WPD is shown in Figure 7. In this paper, the input data *d* denotes the vibration sensory signal collected and its length is *N*. The corresponding wavelet coefficients of *d* in the *j*th level for WPD can be written as [37]:

(12) $$\begin{array}{ll} d_{j}^{n}(k)=\sum_{m}h_{0}(m-2k)d_{j-1}^{n/2}(m) & \left(\text{n is even}\right) \end{array}$$

(13) $$\begin{array}{ll} d_{j}^{n}(k)=\sum_{m}h_{1}(m-2k)d_{j-1}^{(n-1)/2}(m) & \left(\text{n is odd}\right) \end{array}$$

where, *n* is the index of sub-bands, *h_0_* and *h_1_* are a pair of orthogonal filters for decomposition, *h_0_* is low pass filter and *h_1_* is a high pass filter. By decomposing the signal into different frequency band, the influence of noisy disturbance will be weakened obviously. Therefore, component of useful signal at each level can be enhanced correspondingly, which improves the robustness of the extracted features. Here, the sub-band energy $E_{j}^{n}$ at *j*th level are then selected as initial features whose mathematical expression can be calculated as:

(14) $$E_{j}^{n}={\sum_{k}\left(d_{j}^{n}(k)\right)}^{2}$$

As described above, when the decomposed level of WPD is selected to be *u*, the number of the feature vectors is 2*^u^*. The energy feature in each sub-band is calculated according to Equation (12) and the feature set *F* is generated correspondingly as $F=\left\{E_{u}^{1},E_{u}^{2},\ldots ,E_{u}^{2^{u}}\right\}.$

### 3.4. Feature Selection

Irrelevant and redundant features negatively affect the classification performance of the classifier. In this study, a sequential forward selection algorithm named the minimum-redundancy maximum-relevance (mRMR) [38] is employed to search for the optimal feature combination from the original features. The mRMR algorithm utilizes mutual information (MI) to select those optimal features which can best fulfill the minimal redundancy (Min-Redundancy) and maximal relevance (Max-Relevance) criterion.

Within the mRMR algorithm, MI is to estimate the correlation between two variables so as to quantify both the relevance and redundancy. The mathematical expression of MI is given as:

(15) $$I(x,y)=\iint p(x,y)\log\frac{p(x,y)}{p(x)p(y)}\text{dxdy}$$

where *x* and *y* are two vectors, *p*(*x,y*) is their joint probabilistic density, both *p*(*x*) and *p*(*y*) denote the marginal probabilistic densities.

*F* is defined as the original feature set which concludes *h* features and class label *c*. Let *F_a_* denote the already selected feature set with m features, *F_b_* denotes the candidate feature set with *n* features. *m* plus *n* is equal to *h*. Initially, the relevance of the features in *F_b_* set is calculated by:

(16) D=I(f,c)

Then, the redundancy *R* of the feature *f* in *F_b_* with the features *f_j_* in *F_a_* can be calculated by [39]:

(17) $$\begin{array}{cc} R=\frac{1}{m}\sum_{f_{j}\in F_{a}}I(f,f_{j}) & \left(j=1,...,m\right) \end{array}$$

Finally, the maximum relevance and minimum redundancy feature in *F_b_* are selected based on the following rule [39]:

(18) $$\underset{f\in F_{b}}{\max};I(f,c)-\frac{1}{m}\sum_{f_{j}\in F_{a}}I(f,f_{j})=$$

Based on the mRMR criteria, the selected feature is removed from *F_b_* and put into *F_a_*. The new calculation process starts over based on the updated *F_b_* and *F_a_*. Initially, *F_a_* is an empty set (*i.e.*,*m*=0), *F_b_* contains *h* features (*i.e.*,*n*=*h*). The relevance of the features in *F_b_* set is calculated based on Equation (14) and the feature corresponding to the maximum relevance is selected as the first feature and put into *F_a_* set. This feature evaluation process will continue *h*−1 rounds and a rearranged features set *F′* based on mRMR algorithm is obtained as $F^{\prime }=\left\{f_{1}^{\prime },f_{2}^{\prime },\ldots f_{k}^{\prime }\ldots ,f_{h}^{\prime }\right\}$. The first *k* features are then selected and form the final feature set $D=\left\{f_{1}^{\prime },f_{2}^{\prime },\ldots f_{k}^{\prime }\right\}$which are put into DE-EAM based classifier to realize the multi-fault recognition of the rolling element bearing.

## 4. Experimentation and Validation

### 4.1. Data Preparation

**Figure 8.**
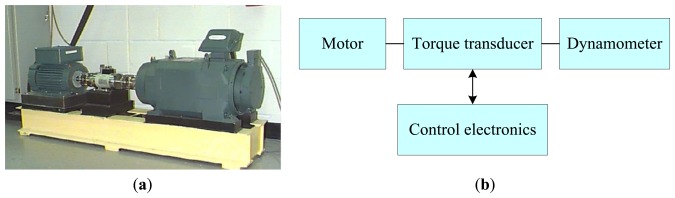
Experiment setup: (**a**) Picture of test rig; (**b**) Framework of test.

To verify the effectiveness of the DE-EAM-based diagnosis method, experimental data of four types of bearing states (normal bearing, inner race fault, ball fault and outer race fault) under different operating loads (1Hp, 2Hp, 3Hp) were collected. The experiment setup of test rig is shown in Figure 8. The deep groove ball bearing (type: 6205-2RS JEM SKF) was utilized to support the motor shaft and some parameters are given as: inside diameter: 0.9843 in; outside diameter: 2.0472 in; ball diameter: 0.3126 in; pitch diameter: 1.537 in. An accelerometer was mounted at the driven end of the motor to collect vibration signals. These different faults were introduced using the electric discharge machining (EDM) method. The sampling frequency was set to be 12,000 Hz because the vibration sensory signal of the rolling bearing is mainly focused on this band.

**Figure 9.**
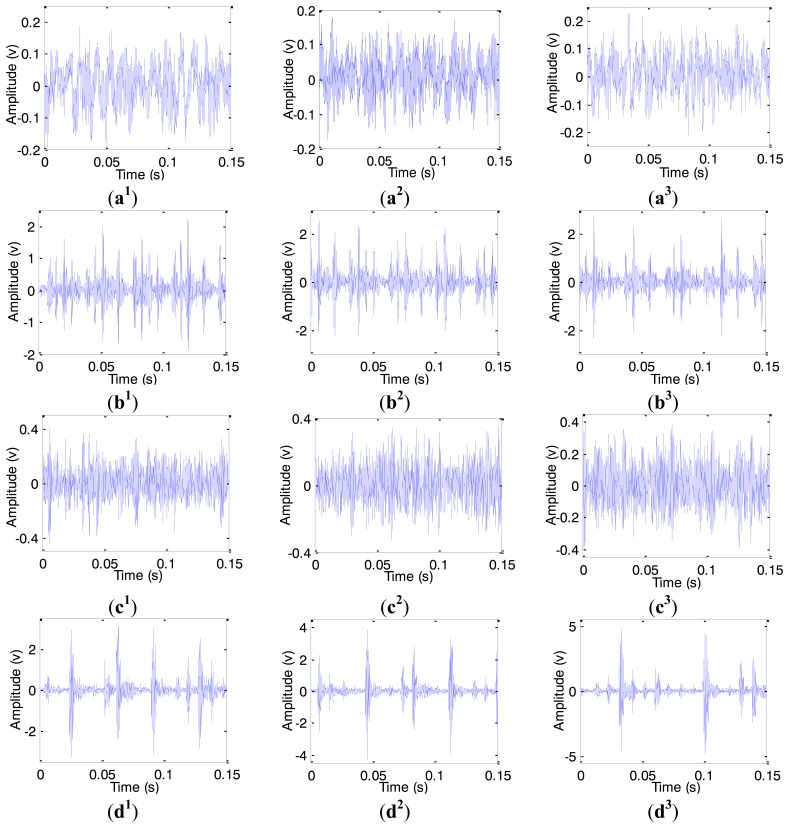
Time domain waveform of fault bearing vibration signal under different loads. (**a**)–(**d**) denote normal bearing, inner race fault, ball fault and outer fault, respectively. The subscript 1, 2 and 3 represent the 1Hp, 2Hp, 3Hp load, respectively.

Each type of bearing was tested under three different loads (1Hp, 2Hp, 3Hp) and 100 samples were collected, respectively. The length of each sample was selected as 2048 which cover more than five cycles of shaft rotating periods. Finally, the number of samples corresponding to each bearing state is 300 and the total number is 1200. Figure 9 shows the time domain waveforms of the four types of rolling bearing under three different loads.

**Figure 10.**
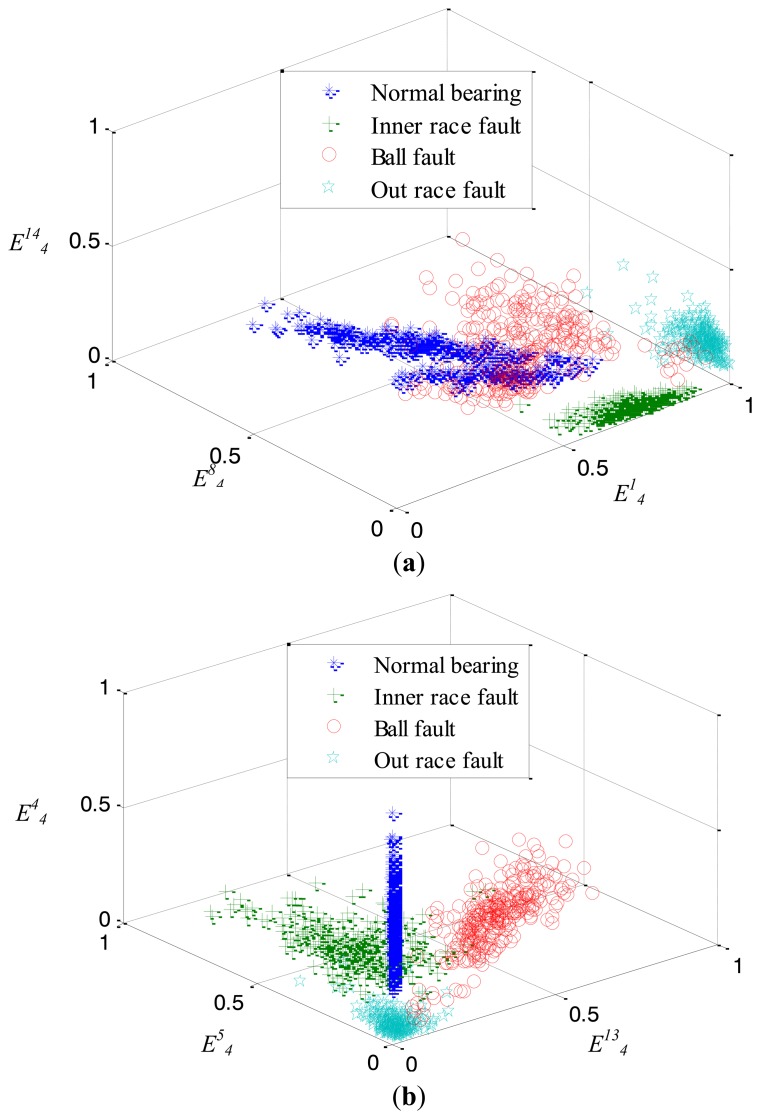
Features spatial distribution of four kinds of bearing states.

**Figure 11.**
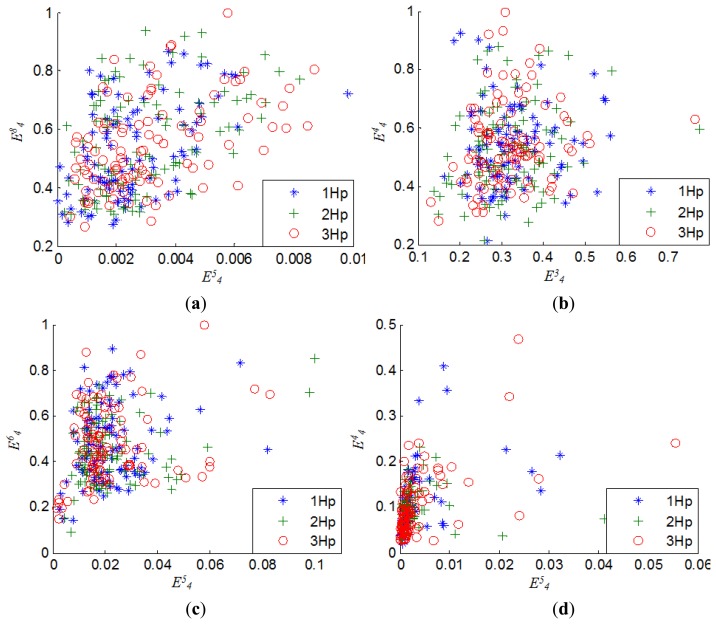
Features spatial distribution for each bearing state under three different loads: (**a**) normal; (**b**) inner race fault; (**c**) ball fault; (**d**) outer race fault.

The WPD is introduced to extract features from the original vibration samples. Here, the decomposed level *u* is selected as 4, which lead to that the dimensionality of the original feature being 16 and a feature set *F* is formed with size 1200 × 16. The mRMR method is then applied to search a salient feature. Here, the target dimension *k* is chosen as 6. After mRMR based feature selection, the first six features $\left\{E_{4}^{1},E_{4}^{4},E_{4}^{13},E_{4}^{5},E_{4}^{8},E_{4}^{14}\right\}$ are selected sequentially from *F* and constructed as the new feature subset *D* whose size is 1200 × 6. Figure 10 shows the features spatial distribution of four kinds of states of rolling element bearings. It can be clearly seen that some feature data of different fault states overlap with each other, which increases the difficulty of the decision boundary representation. In addition, Figure 11 demonstrates the influence of load on the features' spatial distribution for every bearing state. It can be seen that the distribution scopes of the features under different loads are obviously different from each other, although their fault state is the same. In such a case, this casts more difficulty on depicting the spatial distribution of each class and judging the bearing states accurately.

### 4.2. Cross-Validation

Cross-validation is a widely used scheme to evaluate the performance of a classifier. For *v*-fold cross-validation [40], all samples are randomly divided into *v* subsets with equal size and *v* iterations are performed according to the following rule: one different subset is used for validation while the remaining *v*−1 subsets are used for training to construct the classifier model. After *v* iterations, all results are averaged to produce the final estimation of the classifier. The major advantage of cross-validation is that all data are used for both training and validation, and each data is used for validation exactly once. In such case, the accuracy estimation owns a lower variance compared to an accuracy estimate using only one training and validation set. In this paper, 2-fold cross-validation is adopted to separate training samples *D* and evaluate the performance of the classifier.

### 4.3. Parameters Optimization

For the EAM classifier, four network parameters, vigilance parameter *ρ̄*, learning rate *γ*, effective diameter *D_m_*, and ratio of minor-to-major axes lengths *μ* need to be optimized using the DE optimization algorithm. The control parameters in DE are defined as follows: scaling factor *F* = 0.5, crossover rate *C* = 0.2 and the number of population *N_p_* = 40. In addition, the maximum generation number *g*_max_ is selected as 20.

**Figure 12.**
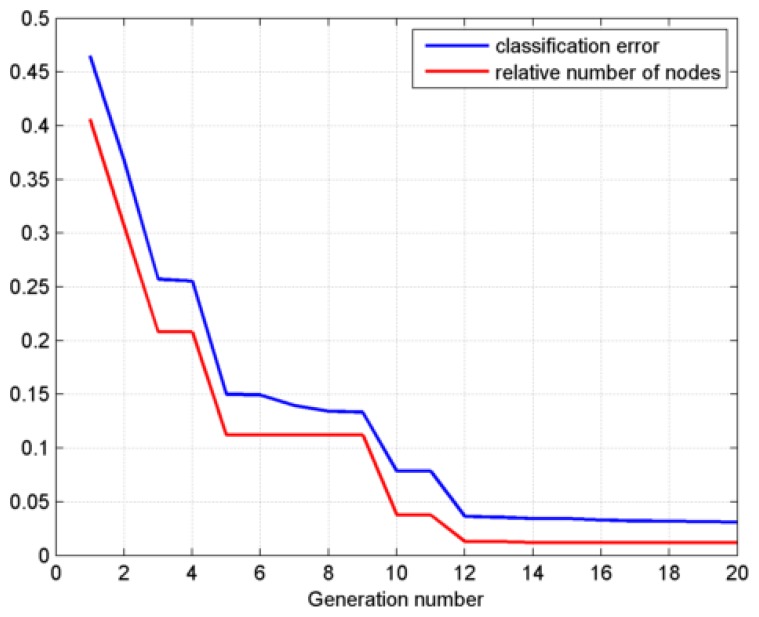
Variation of the fitness functions with the generation number.

The classification error (*Ce*) and relative number of nodes (*R_nod_ =nd*/*M*, where *nd* is the number of EAM nodes, *M* is the dimensionality of samples) are the two most important indicators to evaluate the performance of the EAM classifier. *Ce* directly denotes the classifying ability, the smaller the error, the higher the ability. *nd* represents the computing speed, the less the nodes, the faster the network. In this paper, both *Ce* and *R_nod_* are selected as the fitness function of DE optimization. Figure 12 shows the variation of the two fitness functions with the generation number. It can be seen that both fitness curves tend to be smooth after 12 generations. Therefore, the selection of *g*_max_ is suitable for guaranteeing the convergence of the DE algorithm.

After the maximum generation is satisfied, 40 populations are obtained in which each contains four EAM parameters. To get the final solution, a compound indicator Φ*_t_* is calculated for every possible solution and the final index *i* of the optimized parameters correspond to the minimum Φ*_t_* value, that is:

(19) $$\Phi_{i}=\min\left\lfloor e^{Ce(t)}+e^{R_{\text{nod}}(t)}\right\rfloor (t\in (1,\ldots ,N_{p}))$$

Where *Ce*(*t*) and R*_nod_*(*t*) represent the classification error and relative number of nodes in *t*th population of the objective generation, respectively. After the index *i* is determined based on Equation (17), the final solution vector **Y***_i,g_* which contains four optimized parameters will be utilized to construct the EAM classifier.

### 4.4. Classification and Analysis

**Figure 13.**
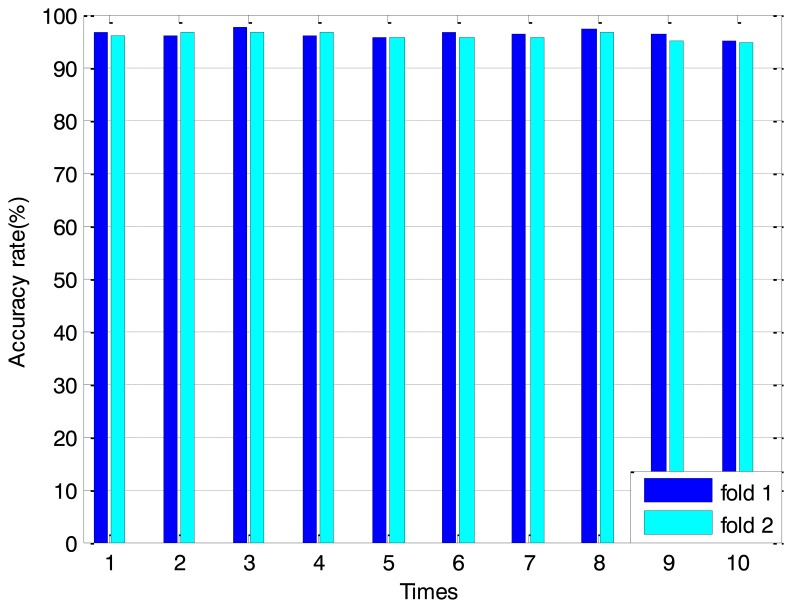
The averaging accuracy of the EAM classifier under different folds and rearrangement orders.

To evaluate the proposed DE-EAM classifier accurately, the order of feature samples is randomly rearranged ten times and a two-fold cross validation method is adopted simultaneously. Figure 13 shows the averaging classification accuracy of the four types of bearing state under every case. In this figure, the *x* axis denotes the repeat times and the *y* axis represents the classification accuracy. It can be obviously seen that the minimum accuracy is 94.75% and the maximum accuracy is 97.5%. The overall averaging classification accuracy can reach 96.1%.

To further show the capability of the DE-EAM classifier, the accuracy of recognizing each kind of fault state is also calculated and listed in Table 1. It can be seen that for the normal bearing, the minimum accuracy is 98% and the maximum is 100%. For the recognizing of the ball pit fault, the lowest accuracy appears, although it can be seen that the average accuracy can still reach 92.9%. Therefore, it can be proven that DE-EAM shows excellent performance for recognizing the fault type of the rolling element bearings.

## 5. Conclusions

In this paper, a novel monitoring system combining an EAM network with a DE optimization algorithm is proposed to realize fault diagnosis of rolling element bearings. Within this framework, the original features are firstly extracted from the vibration sensory signals using WPD to characterize the low frequency and high frequency characteristics of the rolling element bearing. Moreover, the mRMR feature selection method is utilized to reduce the redundancy and irrelevancy of the features vectors. Based on the optimal selected features, the DE-EAM method is proposed to establish the corresponding classifier to realize the diagnosis of the rolling element bearings. The main advantage of the EAM classifier lies in that hyper-ellipsoids are adopted to represent the geometric shape of the feature spatial distribution and the smoothing operation algorithm is used to get a more accurate decision boundary. Moreover, the DE algorithm is integrated with EAM to obtain the optimal network parameters by the limited evolution operations. To verify the robustness and accuracy of the DE-EAM-based system, the samples under four kinds of bearing fault status are collected and organized by random order arrangement and two-fold cross validation strategy. Moreover, the EAM classifier is constructed based on the optimal network parameters which are calculated by the DE optimization. The classification results of four kinds of rolling element bearings show that the overall average accuracy is 96.1% and the maximum average accuracy for recognizing a single fault state can reach 99.8%, which demonstrates that the DE-EAM method can accurately realize fault diagnosis of rolling element bearings.

## Figures and Tables

**Table 1.** The classification accuracy of DE-EAM classifier for each bearing fault state.

**Fault Type**	**Fold Number**	**Times of Random Ranking**										**Average Value**

		**1**	**2**	**3**	**4**	**5**	**6**	**7**	**8**	**9**	**10**	
Normal	1	1	1	1	1	1	1	1	1	1	1	**99.8**
	2	1	1	1	1	0.99	1	1	1	0.99	0.98	

Inner	1	0.94	0.92	0.98	0.92	0.95	0.94	0.97	0.97	0.93	0.92	**93.95**
	2	0.92	0.94	0.96	0.95	0.93	0.90	0.95	0.97	0.93	0.90	

Ball	1	0.94	0.94	0.94	0.94	0.90	0.94	0.90	0.94	0.94	0.90	**92.9**
	2	0.94	0.94	0.93	0.94	0.96	0.94	0.90	0.92	0.90	0.93	

Outer	1	0.98	0.98	0.98	0.98	0.98	0.98	0.98	0.98	0.98	0.98	**97.8**
	2	0.98	0.98	0.98	0.98	0.95	0.98	0.98	0.97	0.98	0.98	
